# Prevalence of primary aldosteronism in acute stroke or transient ischemic attack: a systematic review and meta-analysis

**DOI:** 10.3389/fendo.2024.1355398

**Published:** 2024-03-07

**Authors:** Josephine McCarthy, Mitchell Munnings, Benjamin Clissold, Peter J. Fuller, Jun Yang, Thanh G. Phan

**Affiliations:** ^1^ Department of Medicine, Monash University, Clayton, VIC, Australia; ^2^ Department of Endocrinology, Monash Health, Clayton, VIC, Australia; ^3^ Centre for Endocrinology and Metabolism, Hudson Institute of Medical Research, Clayton, VIC, Australia; ^4^ Department of Endocrinology, Eastern Health, Melbourne, VIC, Australia; ^5^ Department of Endocrinology, Launceston General Hospital, Launceston, TAS, Australia; ^6^ Department of Neurology, Monash Health, Clayton, VIC, Australia

**Keywords:** primary aldosteronsim, hypertension, stroke, transient ischaemic attack, blood pressure

## Abstract

**Background and purpose:**

Primary aldosteronism (PA) is the most common endocrine cause of secondary hypertension with a prevalence of 14% in patients with newly diagnosed hypertension. Patients with PA experience a higher rate of cardiovascular events including stroke when compared to those with blood pressure matched essential hypertension. This systematic review and meta-analysis summarize current evidence on the prevalence of PA in patients with acute stroke or transient ischemic attack (TIA).

**Methods:**

Two reviewers independently reviewed the literature for observational studies on the prevalence of PA in patients with acute stroke or TIA. MEDLINE and Embase were searched for studies up to December 13, 2023.

**Results:**

Three single center studies conducted in Japan, Singapore and China were found to meet the inclusion criteria. The reported prevalence of PA in two cohort studies of adults with stroke or TIA were 3.1% and 4.0% and a third cross-sectional study in adults under 45 years old revealed a prevalence rate of 12.9%. Following a meta-analysis, the pooled prevalence of PA in adults with stroke or TIA is 5.8% [95% CI 1.6%-12.3%].

**Conclusions:**

A considerable proportion of patients with stroke or TIA may have PA as the underlying cause of their hypertension. Given the increased risk of stroke associated with PA, clinicians should consider screening for PA in hypertensive patients with stroke or TIA. Further research is needed to evaluate the effect of timing and interfering medications on test results, which will inform an evidence-based approach to testing for PA following TIA or stroke.

**Systematic review registration:**

https://www.crd.york.ac.uk/PROSPERO/, identifier CRD42022328644.

## 1 Introduction

Primary aldosteronism (PA), also known as Conn’s syndrome, is a potentially curable form of secondary hypertension. PA is characterized by autonomous adrenal production of aldosterone, independent of renin production, resulting in a high aldosterone-to-renin ratio (ARR) (1). Studies in the general hypertensive population have found a PA prevalence of 5-15% (2–4). Hypertension due to PA results in a higher rate of cardiovascular events including stroke when compared with blood pressure (BP) matched essential hypertension. A meta-analysis of 31 studies, including 3838 patients with PA and 9284 patients with essential hypertension found that after a median of 8.8 years from hypertension diagnosis, compared to patients with essential hypertension, patients with PA had an increased risk of stroke with an odds ratio [OR] of 2.58 (95% CI 1.93-3.45) (5). Furthermore, we now know PA is more prevalent in patients with cardioembolic stroke and atrial fibrillation (AF) (6). PA is a treatable condition, either with curative surgery in the setting of a unilateral aldosterone producing adenoma or mineralocorticoid receptor antagonists (MRA) for bilateral adrenal disease. Targeted treatment leads to lower rates of cardiovascular events and stroke (7, 8). Despite the availability of targeted treatment, there is an absence of recommendations to screen for PA in stroke management guidelines (9–12). This systematic review and meta-analysis was performed to answer the clinical research question: what is the prevalence of PA in adults with acute stroke or transient ischaemic attack (TIA)? This evidence will inform future studies and may assist with guideline development.

## 2 Methods

For This systematic review adheres to the PRISMA (Preferred Reporting Items for Systematic Reviews and Meta-Analyses) guidelines (13). The review protocol was registered with the International Prospective Register of Systematic Reviews (PROSPERO) (URL: https://www.crd.york.ac.uk/PROSPERO/; unique identifier: CRD42022328644).

### 2.1 Search strategy

A comprehensive literature search was conducted for the prevalence of PA in acute stroke or (TIA). MEDLINE and Embase were searched using the Ovid platform from inception to December 13, 2023. Three concepts were included in the search strategy: PA, stroke, and TIA. Prevalence was used as a concept in the initial search strategy but then removed due to the few results returned when this term was included. No language or location limits were applied. The online database search was supplemented by a manual search of the reference lists of relevant articles which did not identify any further relevant records. The complete search strategy is provided in **Supplementary Materials**.

### 2.2 Eligibility criteria

#### 2.2.1 Study types

The following study types were considered, cohort, cross-sectional and randomized controlled trials. Studies in languages other than English were considered.

#### 2.2.2 Condition

Assessment of condition, context and population was used to formulate the clinical research question; what is the prevalence of PA in adult patients with acute stroke or TIA? (14) The condition under examination was PA, as diagnosed by a positive confirmatory test with centre-specific diagnostic thresholds (**Table 1**).

**Table 1:** Study Characteristics.

First author, Year, Country	Study design, sample size, n	Stroke, n (%)	TIA, n (%)	Age, y	Male (%)	Interfering medications	Positive ARR threshold	Screen test timing from stroke or TIA	Positive Confirmatory test threshold	PA prevalence in hypertensive patients, % (n)	PA prevalence, % (n)
Miyaji (15), 2016, Japan	Cohort, single center, 427	Ischemic 256 (60), ICH 106 (24.8), SAH 38 (8.9)	27 (6.3)	74^*^	56.7	Yes	ARR ≥200 pg/mL per ng/mL/hr (67 pmol/L per mU/L) and PAC ≥12 ng/dL (≥332 pmol/L)	Admission and 1 week	Rapid ACTH test: ratio of maximal PAC to cortisol ≥8.5	4.9 (14/288) 5.5^‡^	4.0 (17/427) 4.6 (17/373) ^‡^
Nguyen (6), 2022, Singapore	Cohort, single center, 192	Ischemic 156 (81.3), small artery occlusion 50 (26), large artery atherosclerosis 25 (13), cardioembolic 19 (10), undetermined 62 (32.3), hemorrhagic 20 (10.4)	16 (8.3)	58^†^	71	Yes	ARR >277 pmol/l per ng/ml/h (33.7 pmol/L per mU/L)	2-4 months	Seated SIT: post saline PAC >138 pmol/L OR hypokalemia with undetectable PRA and PAC >277 pmol/L	4.0 (95% CI: 0.9% –7.1%) (6/150)	3.1 (6/192) (95% CI: 1.2-6.7%)
Tang (16), 2020, China	Cross-sectional, single center, 116	Ischemic 71 (61), hemorrhagic 41 (35)	5 (4.3)	39^*^	75	No	ARR >1.00 ng/dl per μIU/ml (27.7 pmol/L per mU/L) and PAC >8 ng/dl (>221 pmol/L)	>3 months	Captopril challenge test: 2 hr PAC >11 ng/dl (55pmol/L)	21.2 (14/66)	12.9 (15/116)

ACTH, adrenocorticotropic hormone; ARR, aldosterone to renin ratio; ICH, intracerebral hemorrhage; PA, primary aldosteronism; PAC, plasma aldosterone concentration; PRA, plasma renin activity; SAH, subarachnoid hemorrhage; SIT, Saline Infusion Test; TIA, transient ischaemic attack. *Mean age, †Median age, †Prevalence in the 373 patients who completed both screening tests. Conversion factors: Aldosterone; 1ng/dL =27.7pmol/L, 1ng/dL = 10pg/mL. Renin: plasma renin activity 1 ng/mL/h = direct renin concentration 8.2 mU/L.

#### 2.2.3 Context

The context was adult inpatients or outpatients with acute stroke or TIA.

#### 2.2.4 Population

The population was adults 18 years old and over.

#### 2.2.5 Exclusion criteria

Studies were excluded if 1) they were duplicates, 2) they were case series or case studies, 3) the participants were under the age of 18 years old, 4) it was the wrong patient population, or 4) there was no full text available.

### 2.3 Study selection

Duplicates were removed prior to importing articles into COVIDENCE. Two review authors (JM and MM) independently reviewed titles and abstracts against the eligibility criteria with translation assistance from a colleague fluent in French for one article published in French. Irrelevant articles were removed. The full texts of remaining articles were assessed against eligibility criteria. Any conflicts in study selection between the two review authors (JM, MM) were resolved by the senior review author (JY).

### 2.4 Data extraction

Two reviewers (JM, MM) independently performed two rounds of screening; (1) title and abstract and (2) full text of the remaining studies. COVIDENCE was used to record the extracted data. Data recorded included author, year of publication, study design, participant details, study setting, population characteristics, and results of PA screening and confirmatory tests. Any discrepancies in data recorded by the two reviewers (JM, MM) were resolved by a senior author (JY). The data extraction form is provided in **Supplementary Materials**.

### 2.5 Assessment of risk of bias

The University of Adelaide Joanna Briggs Institute’s (JBI) Critical Appraisal Tool Checklist for Studies Reporting Prevalence Data was used by the two reviewers (JM and MM) to assess methodological quality of studies and risk of bias (14). The following areas of bias were assessed: sample frame, sampling, sample size, description subjects and setting, data analysis coverage, validity of methods to identify condition, statistical analysis and response rate. The complete data collection tool is provided in **Supplementary Materials**.

### 2.6 Statistical analysis

Statistical analyses were performed using Rstudio version 2022.12.0 Build 353 tidyverse and metaphor packages. To account for heterogeneity between studies a random effects model was used. Heterogeneity was assessed using the I^2^ value. Heterogeneity interpretation was guided by the Cochrane Handbook; I^2^ of 0-40% might not be important, 30-60% may represent moderate heterogeneity, 50-90% may represent substantial heterogeneity and 75-100% considerable heterogeneity (17).

### 2.7 Deviations from the protocol

There were no deviations from the systematic review protocol registered on PROSPERO.

### 2.8 Roles and responsibilities

JM developed the search strategy, performed the literature search, screened title and abstracts followed by full text review, data extraction and manuscript preparation. MM independently screened title and abstracts followed by full text review, data extraction and assisted with manuscript preparation. TP and JM performed the meta-analysis. JY assisted with the design of the systematic review and resolved conflicts in article eligibility and data extraction.

## 3 Results

### 3.1 Search results

**Figure 1:**
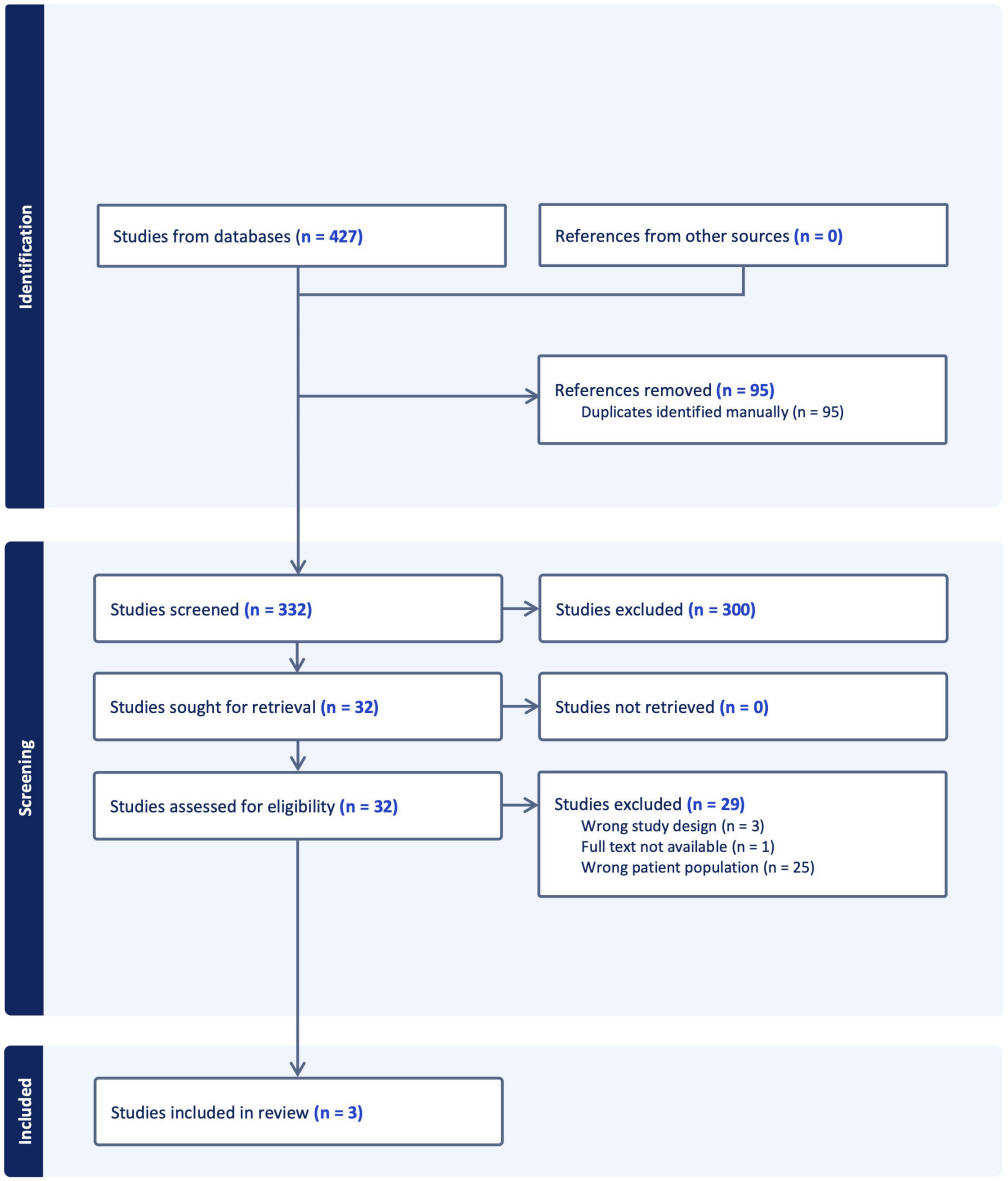
Preferred reporting items for systematic reviews and meta-analyses flowchart of included studies.

The online databases yielded 427 articles; 95 duplicate articles were removed leaving 332 articles imported to COVIDENCE for screening. Following title and abstract screening, 32 full text articles remained for screening. Three articles fulfilled eligibility criteria for inclusion (**Figure 1**) (6, 15, 16).

### 3.2 Study characteristics

Two single center studies, one from Singapore and the other from Japan, were cohort studies of patients admitted with stroke or TIA (6, 15). The third, also a single center study undertaken in China, was a cross-sectional study in young adults with stroke or TIA (16). All studies assessed the prevalence of PA in patients with stroke or TIA. The characteristics of the three included studies are presented in **Table 1**.

### 3.3 Prevalence of PA in patients with stroke or TIA

The prevalence of PA in the prospective cohort study by Miyaji et al. was 4.0% (17 of 427) (15). This was based on two ARR screening tests at initial hospital presentation and 6.8+/- 3.5 days after hospitalization, and the ACTH confirmatory test (**Table 1**). The prevalence of PA amongst patients with a history of hypertension was 4.9%. The mean cohort age was 74.3 years. The criteria for a positive screening test using plasma aldosterone concentration (PAC) and plasma renin activity (PRA) were ARR ≥200 pg/mL per ng/mL/hr (67 pmol/L per mU/L) and PAC ≥12 ng/dL (332 pmol/L) based on the Japan Endocrine Society guidelines. Confirmatory testing was performed if both screening tests were positive. Exceptions were made for some patients with only one positive screening test to proceed to confirmatory testing. This was due to the potential interference of medications given prehospital or during admission which may have resulted in a false negative screening test. Seven patients with an initial negative ARR and a follow up positive ARR went on to confirmatory testing of which two were diagnosed with PA. Five patients with an initial positive ARR and a follow-up negative ARR went on to confirmatory testing of which none were diagnosed with PA.

The prospective cohort study by Nguyen et al. used a single ARR screening test at two to four months post stroke or TIA and found a prevalence of PA of 3.1% (6 of 192) (6). Twenty six of 192 (14%) participants had a positive ARR. In patients with hypertension the prevalence of PA was 4.0% (95% CI: 0.9-7.1%). The median cohort age in this prospective study was 58 years [range 21-78 years]. The criteria for a positive screening test using PAC and PRA was an ARR cut-off >277 pmol/L per ng/ml/h (33.7 pmol/L per mU/L). Of the 192 participants who had an ARR screening test, 14 were positive and underwent a confirmatory saline suppression test of which 3 were positive. Another three patients were diagnosed with PA without confirmatory testing due to elevated aldosterone, undetectable renin and hypokalemia, or a cardiac/renal contraindication to saline suppression test.

In the cross-sectional study by Tang et al. of adults under the age of 45 years old with TIA or stroke, the prevalence of PA was 12.9% (15 of 116) (16). In patients who also had hypertension, the prevalence of PA was 21.2%. The mean age was 39.1 years. The ARR cut-off was 1.00 ng/dl per µIU/ml (27.7 pmol/L per mU/L) based on plasma aldosterone and renin concentrations. Duration between stroke or TIA and tests varied but no patients were screened within the first 3 months. Interfering medications were withdrawn prior to screening and confirmatory testing was performed with the captopril challenge test.

### 3.4 Meta-analysis of prevalence of PA in stroke or TIA

**Figure 2:**
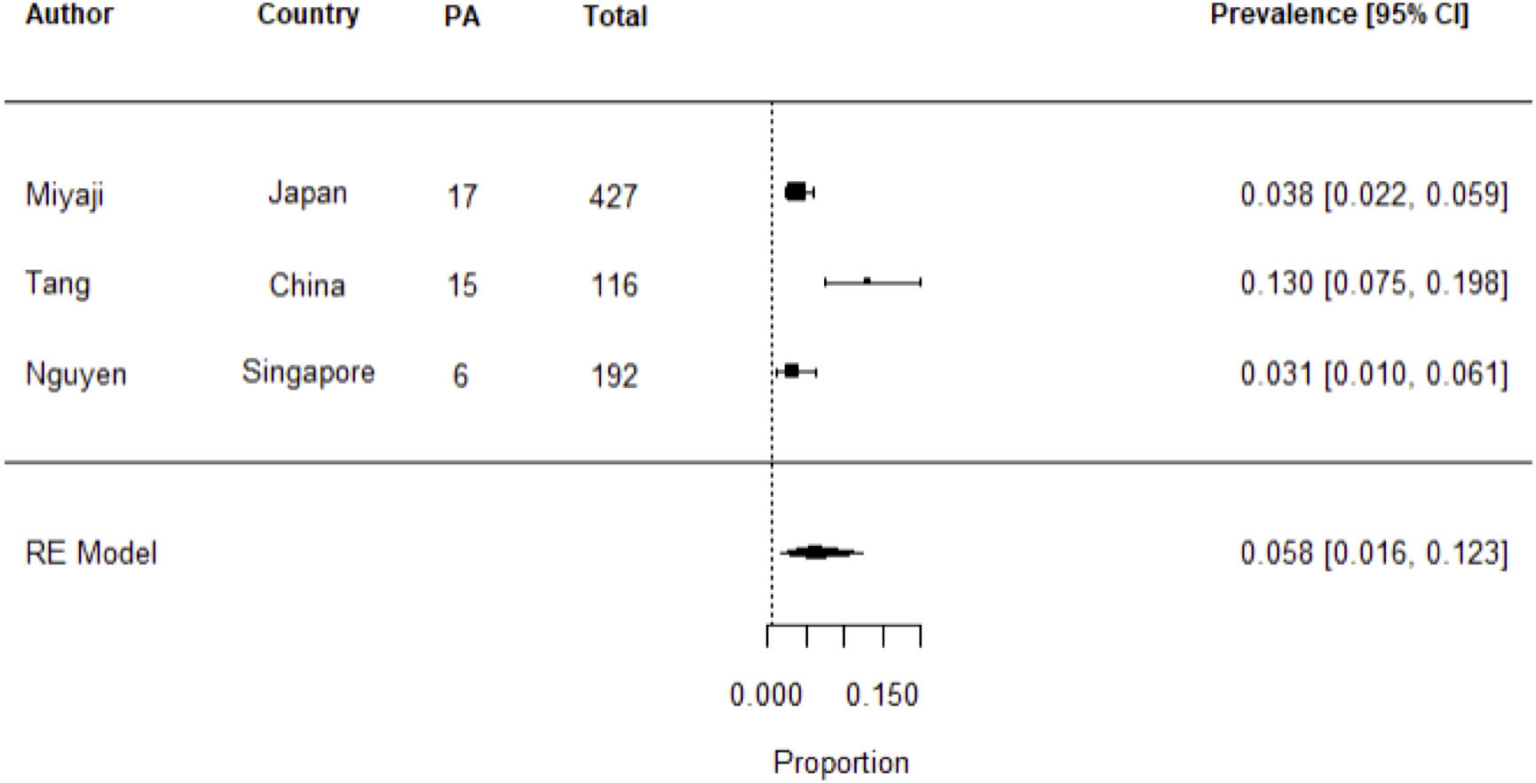
Meta-analysis of studies on the prevalence of PA in stroke or TIA using random effects model. Heterogeneity: Tau^2^ = 0.0085; Q = 12.18; df = 2 (*p* = 0.0023); I^2^ = 87.66%. Test for overall effect: Z = 4.36 (*p* = <0.0001).

The pooled prevalence of PA in patients with stroke or TIA across the three studies was 5.8% [95% CI 1.6%-12.3%], I^2 =^ 87.66% (**Figure 2**). In the presence of heterogeneity, one would normally proceed to a meta-regression analysis, however given there were only three studies, this was not pursued.

### 3.5 Risk of bias assessment of included studies

The three included studies used an appropriate sample frame of patients with TIA or stroke to address the target population. Tang et al. investigated prevalence only in adults <45 years and the smaller sample size may contribute to meta-analysis heterogeneity. All studies described the study subjects and setting in detail and data analysis was conducted with sufficient coverage of the identified sample. Miyaji et al. used the rapid Adrenocorticotrophic Hormone (ACTH) confirmatory test which is not well validated and may result in classification bias. Overall, using the University of Adelaide JBI Critical Appraisal Tool Checklist for Studies Reporting Prevalence Data, the three studies had a low risk of bias.

### 3.6 Management of interfering medications during PA testing in the setting of stroke or TIA

Two thirds of reported studies in this systematic review and meta-analysis (Miyaji et al. and Nguyen et al) continued interfering medications during screening and confirmatory testing. Interfering medications were withdrawn for screening and confirmatory testing in the study by Tang et al. where no patients were screened within the first three months of stroke or TIA (16). However, the prospective cohort studies where patients were assessed in the acute and subacute periods, interfering antihypertensive medications were continued during screening and/or confirmatory testing (6, 15). In Miyaji et al. 55.3% of all patients were on antihypertensive medications (15). In patients with PA, 52.9% (9 of 17) were on antihypertensive medication: 52.9% (9 of 17) were on calcium channel blockers, 11.8% (2 of 17) were on angiotensin converting enzyme inhibitors (ACEI) or angiotensin receptor blockers (ARB) and 5.9% (1 of 17) were on diuretics; no patients were on other antihypertensive medications including beta blockers (15). The total proportion of patients on antihypertensive medication in Nguyen et al. is not reported (6). However, of the 192 patients who completed screening 38% were on a calcium channel blocker, 35.9% were on an ACEI or ARB, 24% were on a beta-blocker, 2% on diuretics and 1% on alpha blockers (6).

### 3.7 Blood pressure in patients with PA and stroke or TIA

Both Miyaji et al. and Tang et al. found mean systolic blood pressure (SBP) during stroke or TIA admission was significantly higher in patients with PA compared to patients without PA (170.9 +/-26.1 mmHg vs 162.0 +/- 31.6 mmHg (*P* 0.012); and 180.0 +/- 30.9 mmHg vs 148.9 +/-29.6 mmHg respectively (*P* 0.002)) (See **Supplementary Table 1**) (15, 16). Miyaji et al. and Tang et al. also found acute admission mean diastolic blood pressure (DBP) was significantly elevated In PA compared to patients without PA (101.8 +/-15.7mmHg vs 89.2 +/- 20.3mmHg (*P* 0.003): and 125.9 +/-24.5mmHg vs 94.1 +/-20.5mmHg (*P* 0.000)) (15, 16).

The prevalence of hypertension in Miyaji et al’s whole cohort (patients with and without PA) was 67.4%, and 82.4% in patients with PA compared to 68.8% in patients without PA. In patients with PA, mean initial BP was consistent with Stage 2 hypertension (mean BP 180/102 mmHg). Patients without PA also had a mean initial BP consistent with Stage 2 hypertension however the BP was not as elevated (mean BP 162/89 mmHg) compared to patients with PA.

Tang et al. recorded BP during initial admission in the cohort of young adults with stroke. The prevalence of hypertension in the whole cohort (patients with and without PA) was 57%. The prevalence of hypertension in patients with PA was 93.3% compared to 51.5% in patients without PA. Patients with PA also had higher a Grade of hypertension; 0% had Grade I hypertension (SBP 140-159 mmHg and/or DBP 90-99 mmHg), 14.3% had Grade II hypertension (SBP 160-179 mmHg and or DBP 100-109 mmHg), and 85.7% had Grade III hypertension (SBP ≥ 180 mmHg and/or DBP ≥ 110 mmHg) (18). Patients without PA had a lower Grade of hypertension; 23.1% Grade I hypertension, 28.8% Grade II hypertension and 48.1% Grade III hypertension (P = 0.00 when compared to patients with PA). This confirms that the grade of hypertension is positively associated with the presence of PA.

Nguyen et al. did not stratify initial admission SBP according to PA status but did so for three months. The prevalence of hypertension in the whole cohort (patients with and without PA) of Nguyen et al. was 78.1%. The prevalence of hypertension in patients with PA was 100%, compared to 77.4% in patients without PA. At three months when stratified by PA status, median SBP in patients with and without PA was not significantly different (145mmHg vs 137mmHg respectively (*P* 0.36)). On the other hand, at three months median DBP was significantly higher in patients with PA compared to patients without PA; 87.0 mmHg [range 84.3, 92.8] vs 80.0mmHg [74.0, 86.0] (*P* 0.011) (6). Patients with PA had a median BP consistent with American Heart Association Stage 2 hypertension (median BP 145/87 mmHg) versus Stage 1 hypertension in patients without PA (median BP 137/80 mmHg) (19). Resistant hypertension was defined as clinic systolic BP ≥140 mmHg, or diastolic BP ≥90 mmHg, while on three antihypertensive medications. Of the whole cohort 0.05% (9 of 192) had resistant hypertension. The prevalence of PA was higher in patients with resistant hypertension, 11.1% (1 of 9), 95% CI: 0.3%–48.3%.

### 3.8 Potassium levels in patients with PA and stroke or TIA

Miyaji et al. and Tang et al. both found that during admission for stroke or TIA, the mean potassium level in patients with PA were significantly lower compared to patients without PA (3.7 +/- 0.4 mmol/L vs 4.1 +/- 0.5 mmol/L (*P* 0.001); and 3.13 +/- 0.50 mmol/L vs 4.01 +/- 0.40 mmol/L (*P* 0.000) respectively) (15, 16). However, serum potassium was similar in Nguyen et al. where only two of six patients with PA had hypokalemia. Serum potassium was not significantly different when stratified by ARR positive versus ARR negative (potassium 3.9 mmol/L [range 3.6, 4.1] compared with 4.0 mmol/L [range 3.7, 4.2] (*P* 0.25 respectively) (6). Nguyen et al. did not stratify potassium results according to the presence or absence of PA (6).

### 3.9 Comorbidities in patients with PA and stroke or TIA

There were several patient groups in which there was a higher prevalence of PA. Nguyen et al. found a higher prevalence of PA amongst patients with both hypertension and atrial fibrillation (30%, 3 of 10), hypertension and hypokalemia (13.3%, 2 of 15), cardioembolic stroke (10.5%, 2 of 19), and age ≤50 years (6.1%, 3 of 49) (6). Tang et al. and Miyaji et al. examined for differences in the prevalence of PA among those with or without diabetes, but no significant difference was found (15, 16).

### 3.10 Possible origin of stroke or TIA

Nguyen et al. and Tang et al. both used the Trial of Org 10172 in Acute Stroke Treatment (TOAST) Classification for stroke aetiology with the five categories being 1) large-artery atherosclerosis, 2) cardioembolism, 3) small-vessel occlusion, 4) stroke of other determined etiology, and 5) stroke of undetermined aetiology (20). There was no significant difference between the stroke subtypes in patients with PA compared to patients without PA in the studies by Nguyen et al. and Tang et al. (P 1.0 and P 0.674 respectively). Miyaji et al. did not report on aetiology of stroke.

## 4 Discussion

Hypertension is a major modifiable risk factor for ischemic and hemorrhagic stroke (21). Despite PA being the most common endocrine cause of secondary hypertension with an increased risk of AF, stroke and TIA compared to patients with BP matched essential hypertension, there is a lack of research on its prevalence and management in acute stroke or TIA (5). Studies over the last decade have reported a PA prevalence of 5-15% in the general hypertensive population (2–4). Based on 738 patients from three studies, this meta-analysis found a PA pooled prevalence of 5.8% in patients with stroke or TIA.

The sex distribution of PA in this meta-analysis was consistent with that previously reported, where approximately half were men (2). Nguyen et al. and Tang et al. both reported a similar sex distribution of PA in stroke or TIA (66.7% in both studies) (6, 16). Miyaji et al. had fewer men in the cohort (56%) than Nguyen et al. and Tang et al, which may explain the lower proportion of men with PA (29.4%). There were no sub analyses of the sex distribution of PA in patients with TIA.

Difference in patients with PA compared to patients without PA are striking for the higher systolic BP (170-180mmHg vs 148-162mmHg), and lower serum potassium (3.1-3.7mmol/L vs 4.0-4.1mmol/L) in patients with PA. This is consistent with PA being more likely to cause resistant hypertension and hypokalaemia (1). Blood pressure was taken 3 months post stroke with a trend for a higher number of antihypertensive agents in patients with PA compared to without PA (1.7 vs 1.0 respectively) which may account for the lack of difference in SBP between groups in Nguyen et al. (6) The markedly higher DBP in patients with PA compared to without PA (125.9mmHg vs 94.1mmHg) in the younger cohort of Tang et al. has previously been seen in PA when stratified by age (16, 22). There was also a higher prevalence of PA in people with a history of hypertension. Only a small proportion of people were diagnosed with PA but without hypertension (0% - 17.6%). Clinical presentation of stroke or TIA with elevated blood pressure may be a useful trigger for PA testing.

Meta-analyses of prevalence often have high statistical heterogeneity. This meta-analysis displayed high heterogeneity with an I^2^ statistic of 87.6%. A study of 134 meta-analyses of prevalence revealed the median I^2^ was 96.9% (IQR 90.5–98.7) (23) with larger I^2^ in meta-analysis with a higher number of studies or extreme pooled estimates (defined as <10% or >90%). The high I^2^ observed in the present meta-analysis is consistent with other prevalence studies with an extreme pooled prevalence <10%, in this case 5.8%. There are several prevalence modifying factors which contribute to the statistical heterogeneity in this study including differences in age of the populations sampled, hypertension prevalence, ARR threshold, confirmatory tests used and time from stroke to testing. Tang et al. had a younger population and higher prevalence of hypertension. Monticone et al. demonstrated in a prospective cohort study that the prevalence of PA increases with an increase in the stage of hypertension of the cohort (24). Uniformity in population sampling and testing methods may reduce heterogeneity of future meta-analyses.

Time from acute cerebral event to testing for PA differed in the three studies and is likely to impact reported prevalence. Miyaji et al. tested in the first week of the acute cerebral event which may lead to a false negative ARR (25). Miyaji et al. found the acute phase ARR was less reliable at predicting PA than the post stroke ARR, however the evidence is limited by the lack of confirmatory testing in every patient with either one or two positive ARRs (15). Nguyen et al. and Tang et al. tested ARR several months after the acute cerebral event, this subacute time frame for testing may be less prone to false negative ARR. A prospective human study on RAAS during acute ischaemic stroke found that angiotensin I, renin and aldosterone were significantly lower, angiotensin II was unchanged, and angiotensin converting enzyme activity was higher in the acute phase (within 48 hours) compared to post-stroke (8 months) (25). Acute stroke can affect ARR by various mechanisms including, dehydration which increases renin and aldosterone, high BP during the acute stroke phase with higher adrenaline and cortisol levels which lowers aldosterone and renin and interfering antihypertensive medications (25). Further research is required to establish the optimal ARR threshold for PA testing in the context of the acute hormonal changes during acute stroke.

The main limitation for PA testing in someone with established hypertension is the confounding effect of antihypertensive drugs. The Endocrine Society recommends a washout of all interfering antihypertensive medications with the use of substitute medications that have minimal effect on ARR but acknowledges that the ARR can be interpreted accordingly if medication switching is not feasible (1). In this systematic review, only Tang et al. adjusted antihypertensive agents for testing. To maintain stringent BP control during and shortly after stroke or TIA, both Miyaji et al. and Nguyen et al. continued interfering medications through the testing period. The frequent use of antihypertensive agents which can cause false negative screening results may contribute to the lower prevalence of PA in these two cohorts (6, 15). Of note, Nguyen et al. adopted a lower ARR threshold of >277 pmol/l per ng/ml/h (33.7 pmol/L per mU/L) to reduce the number of false negative results caused by interfering medications, although the evidence base for the threshold was not stated. However, only in the absence of medication interference, an ARR cut-off of >70 pmol/mU has a sensitivity and specificity greater than 95% (26). There is a paucity of data to provide any robust recommendation on ARR thresholds whilst on interfering medications (27–29).

Changing antihypertensive medications to non-interfering agents for PA screening can be challenging in the acute stroke period. For patients on ACEI’s, the aldosterone to angiotensin II ratio may be a more reliable marker of aldosterone excess than ARR (30). However, the assay to measure angiotensin II is not widely available and angiotensin II concentration is not reported in any of the three articles. Further research is needed to understand the utility of angiotensin II for the diagnosis of PA in patients with stroke or TIA.

Although ARR was the screening test of choice for each of the three studies, the number of positive ARR required to proceed to confirmatory testing differed. The reported prevalence of PA in Miyaji et al. was 4.0% (17 of 427) however, 26 patients did not have initial blood sampling and 28 patients did not have follow up blood sampling leaving 373 patients with both screening tests. If only patients with both screening tests were considered, the prevalence becomes 4.6% overall and 5.5% in those with hypertension (15). Nguyen et al.’s prospective cohort study found a slightly lower prevalence of 3.1% amongst all patients despite a lower ARR cut-off than Miyaji’s study (6, 15). Of note, in Nguyen et al, more than 50% of the participants with an abnormal ARR had hypokalemia (15 of 26). Previous studies show approximately 30% of patients with PA have hypokalemia, therefore it may be possible that some patients with PA and normokalemia were missed, rendering the prevalence rate an underestimate (1).

The three included studies used different confirmatory tests. Tang et al. and Nguyen et al. used captopril challenge test and saline suppression test respectively, which are two of the four recommended confirmatory tests of the Endocrine Society (1, 6, 16). Miyaji et al. used the rapid adrenocorticotropic hormone (ACTH) test which is not one of the tests recommended by the Endocrine Society (1, 15). A recent systematic review and meta-analysis, limited to English, on the performance of PA confirmatory tests did not find any studies on the validity of the rapid ACTH test (31). Only one study, published in Japanese, has compared the validity of the rapid ACTH test against the captopril challenge test or furosemide plus upright test and found the rapid ACTH test had a sensitivity and specificity of >95% (32). Clinical practice differs internationally for the preferred confirmatory test; this will impact the reported prevalence in each study.

The link between mineralocorticoids and stroke has been established experimentally in rodents (33, 34). Rocha et al. studied Stroke Prone Spontaneously Hypertensive (SHRSP) rats treated with either placebo or the mineralocorticoid antagonist eplerenone for 19 weeks (33). Blood pressure was equally raised in both groups. Placebo treated rats showed clinical signs of stroke earlier and all died by 18 weeks. By comparison, only one eplerenone treated rat showed signs of stroke and died at 18 weeks. Histopathology revealed severe ischemic and hemorrhagic stroke lesions in the placebo treated rats compared with mild cerebral injury in the eplerenone treated rats. Dorrance et al. induced stroke experimentally (thread-occlusion technique) in SHRSP rats of which half were treated with spironolactone and Wistar-Kyoto (WKY) normotensive rats which were treated with placebo (34). Spironolactone reduced cerebral ischemic damage by 50% in SHRSP rats. Dorrance and Rocha both found that despite the reduction in cerebral ischemia following treatment with a mineralocorticoid antagonist, there was no effect on SBP. Limited human studies have established several mechanisms for aldosterone’s adverse vascular effects including endothelial dysfunction, increased arterial wall stiffness, structural cardiac remodeling through atrial dilatation and fibrosis, and electrical remodeling through arrhythmogenicity (35–38). Nguyen et al. found patients with PA had a larger left atrial volume index, which predisposes to AF. Nguyen et al. also found the prevalence of PA in patients with stroke, hypertension and AF was 30%, similar to that found by Seccia et al. in patients with hypertension and AF (42%) (39). Milliez et al. found a 12-fold higher risk of AF in patients with PA compared to patients with essential hypertension (40). The results of these rodent studies confirm aldosterone has harmful actions on cerebral vasculature independent of its ability to increase BP and that treatment with a mineralocorticoid receptor antagonist such as spironolactone or eplerenone can reduce the frequency and severity of stroke.

To summarize, the underlying pathophysiological mechanisms leading to an increased risk of stroke has been explored in rodent studies and limited human studies, it goes beyond that purely due to aldosterone-mediated hypertension. These studies provide compelling evidence that timely diagnosis and targeted treatment can significantly reduce the severity of cerebral injury and the occurrence of stroke.

## 5 Conclusion

This systematic review and meta-analysis revealed that 3.1-12.9% of patients with acute stroke or TIA have PA, with a higher prevalence of up to 22% if only hypertensive patients are considered. However, there was significant variability between studies including the timing of the test, nature of confirmatory testing and medication use. A larger prospective study where patients are screened both in the acute and outpatient settings would help to inform the optimal timing and conditions of PA testing in patients with stroke and TIA. Furthermore, all three studies were in East Asian and South East Asian populations; data from other populations are needed. Additional research will facilitate the development of evidence-based guidelines for PA testing in patients with stroke or TIA so that this highly modifiable cardiovascular risk factor can be efficiently ameliorated.

## Data availability statement

The original contributions presented in the study are included in the article/**Supplementary Material**. Further inquiries can be directed to the corresponding author.

## Author contributions

JM: Conceptualization, Writing – original draft, Writing – review & editing. MM: Writing – review & editing. BC: Writing – review & editing. PF: Writing – review & editing. JY: Conceptualization, Writing – review & editing. TP: Conceptualization, Writing – review & editing.
