# Supplementary material for: Prevalence of primary aldosteronism in acute stroke or transient ischemic attack: a systematic review and meta-analysis
Source: Front Endocrinol (Lausanne). 2024 Mar 7;15:1355398. doi: 10.3389/fendo.2024.1355398 (PMC10956081; doi:10.3389/fendo.2024.1355398)
Supplement: Supplementary file 1 [file DataSheet_1.doc]

Supplemental Material

1. **Detailed Methods**

**Search strategy**

| 1 | primary hyperaldosteronism/ | 7,666 |
| --- | --- | --- |
| 2 | (aldosteronism or hyperaldosteronism).ti,ab. | 10,886 |
| 3 | (conn* adj syndrome).ti,ab. | 934 |
| 4 | (aldosterone adj2 adenoma).ti,ab. | 1,503 |
| 5 | 2 or 3 or 4 | 11,771 |
| 6 | 1 or 5 | 13,210 |
| 7 | exp cerebrovascular accident/ | 267,076 |
| 8 | [stroke.mp](http://stroke.mp/). [mp=title, abstract, heading word, drug trade name, original title, device manufacturer, drug manufacturer, device trade name, keyword heading word, floating subheading word, candidate term word] | 517,073 |
| 9 | (cerebr* adj3 (insult or injur* or accident or insufficiency or arrest)).mp. [mp=title, abstract, heading word, drug trade name, original title, device manufacturer, drug manufacturer, device trade name, keyword heading word, floating subheading word, candidate term word] | 274,879 |
| 10 | ((cerebral or brain) adj2 isch?emia).mp. [mp=title, abstract, heading word, drug trade name, original title, device manufacturer, drug manufacturer, device trade name, keyword heading word, floating subheading word, candidate term word] | 170,440 |
| 11 | (brain adj2 ("vascular accident*" or insult or disturbance or insult*)).mp. [mp=title, abstract, heading word, drug trade name, original title, device manufacturer, drug manufacturer, device trade name, keyword heading word, floating subheading word, candidate term word] | 2,600 |
| 12 | (CVA or CVAS).ti,ab. | 7,696 |
| 13 | 8 or 9 or 10 or 11 or 12 | 667,859 |
| 14 | 7 or 13 | 667,859 |
| 15 | transient ischemic attack/ | 44,042 |
| 16 | ((transient or brain or attack) adj2 isch?emi*).mp. [mp=title, abstract, heading word, drug trade name, original title, device manufacturer, drug manufacturer, device trade name, keyword heading word, floating subheading word, candidate term word] | 208,674 |
| 17 | (TIA or TIAs).ab,ti. | 21,966 |
| 18 | 16 or 17 | 216,637 |
| 19 | 15 or 18 | 216,637 |
| 20 | 14 or 19 | 689,207 |
| 21 | 6 and 20 | 319 |

**Figure S1. Embase search strategy**

| 1 | Hyperaldosteronism/ | 8,412 |
| --- | --- | --- |
| 2 | (aldosteronism or hyperaldosteronism).ti,ab. | 6,844 |
| 3 | (Conn* adj syndrome).ti,ab. | 594 |
| 4 | (aldosterone adj2 adenoma).ti,ab. | 947 |
| 5 | 2 or 3 or 4 | 7,453 |
| 6 | 1 or 5 | 10,263 |
| 7 | exp stroke/ | 158,576 |
| 8 | [stroke.mp](http://stroke.mp/). [mp=title, abstract, original title, name of substance word, subject heading word, floating sub-heading word, keyword heading word, organism supplementary concept word, protocol supplementary concept word, rare disease supplementary concept word, unique identifier, synonyms] | 302,810 |
| 9 | (cerebr* adj3 (insult or injur* or accident or insufficiency or arrest)).mp. [mp=title, abstract, original title, name of substance word, subject heading word, floating sub-heading word, keyword heading word, organism supplementary concept word, protocol supplementary concept word, rare disease supplementary concept word, unique identifier, synonyms] | 19,083 |
| 10 | ((cerebral or brain) adj2 isch?emia).mp. [mp=title, abstract, original title, name of substance word, subject heading word, floating sub-heading word, keyword heading word, organism supplementary concept word, protocol supplementary concept word, rare disease supplementary concept word, unique identifier, synonyms] | 80,667 |
| 11 | (brain adj2 ("vascular accident*" or insult or disturbance or insult*)).mp. [mp=title, abstract, original title, name of substance word, subject heading word, floating sub-heading word, keyword heading word, organism supplementary concept word, protocol supplementary concept word, rare disease supplementary concept word, unique identifier, synonyms] | 1,676 |
| 12 | (CVA or CVAS).ti,ab. | 2,761 |
| 13 | 8 or 9 or 10 or 11 or 12 | 356,463 |
| 14 | Ischemic Attack, Transient/ | 21,348 |
| 15 | ((transient or brain or attack) adj2 isch?emi*).mp. [mp=title, abstract, original title, name of substance word, subject heading word, floating sub-heading word, keyword heading word, organism supplementary concept word, protocol supplementary concept word, rare disease supplementary concept word, unique identifier, synonyms] | 100,677 |
| 16 | (TIA or TIAs).ab,ti. | 8,936 |
| 17 | 15 or 16 | 103,468 |
| 18 | 14 or 17 | 103,468 |
| 19 | 13 or 18 | 372,171 |
| 20 | 6 and 19 | 107 |

**Figure S2. Medline search strategy**

1. **Supplemental Figures**


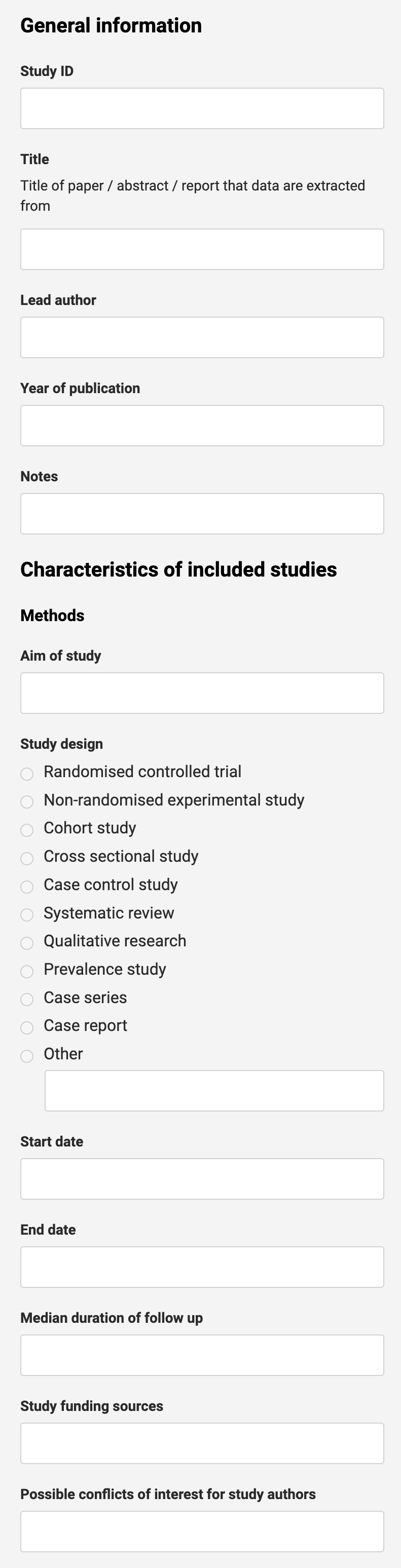

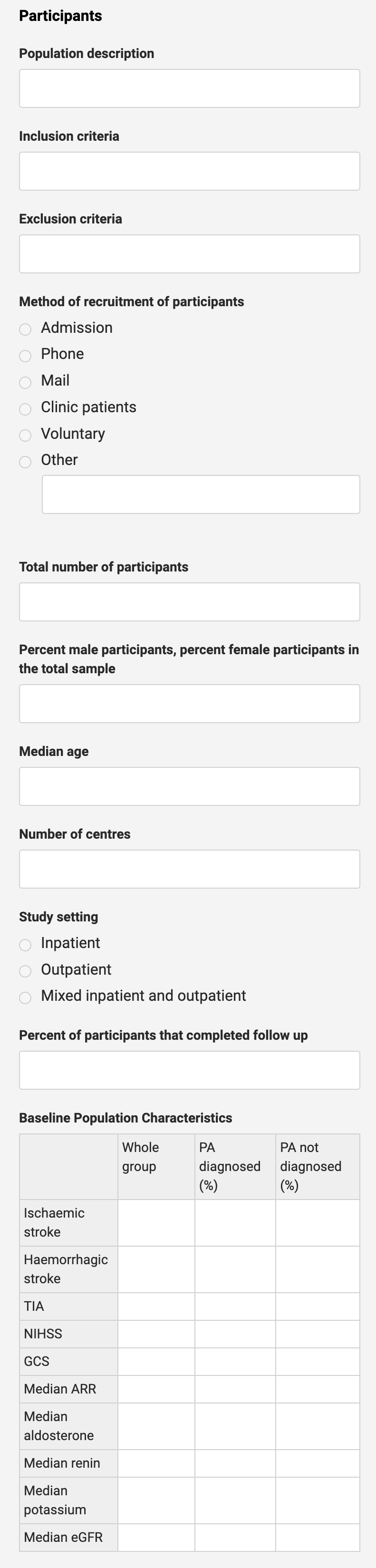

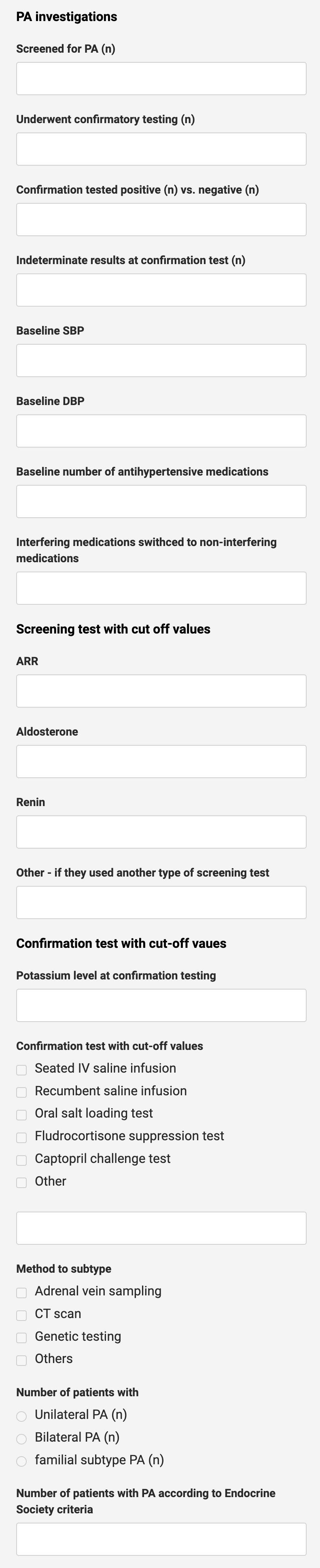
Figure S3. COVIDENCE data extraction template

**JBI Critical Appraisal Checklist for Studies reporting prevalence data**

Reviewer______________________________________ Date_______________________________

Author_______________________Year_________ Record Number_________

|  | Yes | No | Unclear | Not applicable |
| --- | --- | --- | --- | --- |
| 1. Was the sample frame appropriate to address the target population? | □ | □ | □ | □ |
| 1. Were study participants sampled in an appropriate way? | □ | □ | □ | □ |
| 1. Was the sample size adequate? | □ | □ | □ | □ |
| 1. Were the study subjects and the setting described in detail? | □ | □ | □ | □ |
| 1. Was the data analysis conducted with sufficient coverage of the identified sample? | □ | □ | □ | □ |
| 1. Were valid methods used for the identification of the condition? | □ | □ | □ | □ |
| 1. Was the condition measured in a standard, reliable way for all participants? | □ | □ | □ | □ |
| 1. Was there appropriate statistical analysis? | □ | □ | □ | □ |
| 1. Was the response rate adequate, and if not, was the low response rate managed appropriately? | □ | □ | □ | □ |

Overall appraisal: Include □ Exclude □ Seek further info □

Comments (Including reason for exclusion)

**Figure S4. JBI Critical Appraisal Checklist for Studies reporting prevalence data**

1. **Supplemental Tables and Supporting information**

| First author | SBP non PA, mmHg, mean ± SD, median [min, max] | SBP in PA, mmHg, mean ± SD, median [min, max] | DBP non PA, mmHg, mean ± SD, median [min, max] | DBP in PA, mmHg, mean ± SD, median [min, max] | Aldosterone level in PA, median pmol/L | Renin level in PA, median mU/L | ARR in PA, median pmol/L per mIU/L | Potassium in PA, mean ±SD, mmol/L |
| --- | --- | --- | --- | --- | --- | --- | --- | --- |
| Miyaji^14^ | 162.0 +/- 31.6 | 179.9 ± 26.1 | 89.2 +/- 20.3 | 101.8 ± 15.7 | Not reported | Not reported | Not reported | 3.7 ± 0.4 |
| Tang^15^ | 148.9 +/-29.6 | 180.0 ± 30.9 | 94.1 +/- 20.5 | 125.9 ± 24.5 | 639 (IQR 597, 1195) | 2.40 (IQR 2.08, 10.77) | 266 (IQR 57, 5955) | 3.13 ± 0.50 |
| Nguyen^4^ | 137 [126, 149] | 145 [138, 147] | 80.0 [74.0, 86.0] | 87.0 [84.3 – 92.8] | 416 (271, 521) | 0.08 (0.07, 0.14) | 343 (319, 609) | 3.78 ± 0.55 |

**Table S1. Characteristics of patients without primary aldosteronism and with primary aldosteronism**

Abbreviations: ARR = aldosterone to renin ratio; DBP = diastolic blood pressure; IQR = Interquartile range; PA = primary aldosteronism; SBP = systolic blood pressure

Conversion factors: Aldosterone; 1ng/dL = 27.7 pmol/L, 1ng/dL = 10pg/mL. Renin: plasma renin activity 1 ng/mL/h = direct renin concentration 8.2 mU/L
